# Comprehensive Property Investigation of Mold Inhibitor Treated Raw Cotton and Ramie Fabric

**DOI:** 10.3390/ma13051105

**Published:** 2020-03-02

**Authors:** Zhixin Zhao, Wei Cai, Lei Song, Xiaowei Mu, Yuan Hu

**Affiliations:** 1 State Key Laboratory of Fire Science, University of Science and Technology of China, Hefei 230026, China; cai1992@mail.ustc.edu.cn (W.C.); leisong@ustc.edu.cn (L.S.); 2 School of Chemistry and Materials Science, University of Science and Technology of China, 96 Jinzhai Road, Hefei 230026, China; zzx430@mail.ustc.edu.cn

**Keywords:** cotton, ramie, wettability, thermal conductivity, combustion characteristic

## Abstract

At present, research rarely focuses on side effects of the use of mold inhibitors on raw cotton and ramie fabric. Four different mold inhibitors (dimethyl fumarate (DMF), ethyl p-hydroxybenzoate (EHB), propyl p-hydroxybenzoate (PHB), and calcium sorbate (CS)) were used to treat raw cotton and ramie fabric through a dipping method. The optical properties, wettability, thermal conductivity, thermal stability, and combustion properties of treated cotton and ramie samples have been investigated. The reflectance of UV light was improved by the addition of mold inhibitors. In addition, the presence of EHB, PHB, and CS improved the wettability of raw cotton and ramie fabric. It was found that thermal conductivity was slightly increased, influencing the heat insulation effect of the fabrics. Since the additives are flammable, the presence of DMF, EHB, and PHB caused an increase in pHRR and THR for combustion of cotton samples. This addition of CS caused a decrease in pHRR and THR of cotton due to the flame retardancy of CS. This comprehensive investigation of the properties of raw cotton and ramie fabrics treated with these materials should provide a basis for the choice of mold inhibitors.

## 1. Introduction

Cotton has been among the most important agricultural products. It can be processed into a large variety of fabrics applied in the clothing and furniture industries, due to good mechanical properties and affinity to human skin. Ramie fiber is traditional fiber widely used in ancient China which is getting more and more attention from all over the world for its excellent strength, hygroscopicity, and breathability.

Since a huge amount of cotton and ramie are produced, transported, and warehoused every year, mildew prevention measures are of vital importance to avoid unexpected loss. A variety of mold-inhibitors are applied to cotton and ramie fabric to keep it free of mildew [1,2,3]. The main types of mildew preventives include propionic acid and its salts, [4] sorbic acid and its salts, [5] methyl fumarate [6], and p-hydroxybenzoate compounds [7].

Fire prevention of cotton and ramie is also very important since cotton or ramie bales of high density are at risk of burning [8]. Fire accidents in cotton storage occur every year, causing economic and environmental loss. However, some of the mold inhibitors could be the cause of accidents in cotton and ramie storage and transportation due to extreme toxicity and flammability. Thermal gravimetric analysis (TGA) was employed to investigate the thermal decomposition behavior of the chitosan phosphate, butaneteracarboxylic acid, and TiO_2_ nanoparticle treated cotton fabrics [9]. However, other works about the influence of these mold inhibitors on the fire safety of cotton and ramie fabric is rarely reported. 

Fabric gloss is one of the main indicators of the visual style of a fabric, and it is an important basis for evaluating the quality of appearance. Many modification methods using chemical agents have been done to change or improve the luster of textiles [10]. Optical brightness of raw cotton and bleached fabrics is an important performance characteristic. Otherwise, wettability and thermal conductivity are other basic properties of raw cotton and ramie fabrics, [11,12,13] which are closely related to the comfort and durability of their products for wearing. Antimicrobial, UV resistant, and thermal comfort properties of chitosan- and Aloe vera-modified cotton woven fabric have been investigated [14]. Silver nanoparticles have been used to treat textiles and the morphological, antimicrobial, durability, and physical properties of these additives have been studied [15]. The effect of various metallic salts on antibacterial activity and physical properties of cotton fabrics have been investigated [16]. The antibacterial and physical properties of knitted cotton fabrics treated with antibacterial finishes have been examined [17].

Ethyl p-hydroxybenzoate and propyl p-hydroxybenzoate, also known as parabens, are mainly used as preservatives in soy sauce, jams, and refreshing beverages. These are colorless crystal or white crystalline powders and are tasteless and odorless. The preservative effect is better than that of benzoic acid and its sodium salt [18]. With the characteristics of high efficiency and wide-ranging antibacterial, dimethyl fumarate is an effective mold inhibitor [19]. Sorbic acid and its salts have been widely used in preservation of food due to their physiological inertness, their effectiveness even in the weakly acid pH range, and their neutral taste [20,21]. However, the potential influence of these mold inhibitors on these properties influencing storage of cotton and ramie fabrics has rarely been studied.

Herein, four of the most commonly used mold inhibitors (dimethyl fumarate, ethyl p-hydroxybenzoate, propyl p-hydroxybenzoate, and calcium sorbate) were chosen to treat raw cotton and ramie fabric. The optical properties, wettability, thermal conductivity, thermal properties, and flammability of treated cotton and ramie fabric have been assessed.

## 2. Materials and Methods

### 2.1. Materials

Cotton was provided by Zhengzhou Cotton and Jute Engineering Technology and Design Research Institute (Zhengzhou, China, fiber length of 25–35 mm, fineness of 1.56–2.12 dtex, average Rd of 76.8%). Bleached ramie fabric was purchased from Hunan Huasheng Dongting Ramie Company (Hunan, China, 36 × 36 / 79 × 60, 2.9 oz/y^2^). Dimethyl fumarate, ethyl p-hydroxybenzoate, propyl p-hydroxybenzoate, and calcium sorbate were directly obtained from Sigma-Aldrich (Shanghai, China).

### 2.2. Anti-Mildew Treatment of Cotton and Ramie

Dimethyl fumarate (5 g) was dissolved into 500 mL ethanol in a beaker with stirring for 0.5 h. Ethyl p-hydroxybenzoate and propyl p-hydroxybenzoate were dissolved in the same way. Calcium sorbate was dissolved into 500 mL water in a beaker with stirring for 0.5 h. Cotton (120 g) was divided equally into four parts, each of which was immersed into one of the prepared solutions for 24 h, respectively. Then, the cotton was taken out and dried in an oven at room temperature. The samples were denoted as cotton/dimethyl fumarate (cotton/DMF), cotton/ethyl p-hydroxybenzoate (cotton/EHB), cotton/propyl p-hydroxybenzoate (cotton/PHB), and cotton/calcium sorbate (cotton/CS). In addition, bleached ramie fabric was cut into square pieces of 100 × 100 mm. The average thickness and weight of each piece were 0.05 mm and 1.2 g. Then, bleached ramie fabric was immersed into the above solutions for 24 h. The post-processing of ramie was similar to that of cotton. The obtained ramie fabric was denoted as ramie/dimethyl fumarate (ramie/DMF), ramie/ethyl p-hydroxybenzoate (ramie/EHB), ramie/propyl p-hydroxybenzoate (ramie/PHB), and ramie/calcium sorbate (ramie/CS), respectively. Control samples of cotton and ramie were treated by the same procedure without any mold inhibitors in water.

### 2.3. Characterization and Measurement

UV-Vis diffuse reflectance spectroscopy was used to investigate the optical property of cotton fabric. The UV-Vis diffuse reflectance absorption spectra of cotton and ramie were recorded using a SOLID 3700 UV-VIS-NIR spectrophotometer (Shimadzu Corporation, Kyoto, Japan) with a diffuse reflectance accessory (DRA-2500).

Contact angle was determined using a SL200B Contact Angle System (Solon Tech. Co., Ltd., Shanghai, China) at ambient temperature to study the wettability of cotton and ramie fabric. The shooting speed of the camera was 2.5 frames/s. At least six specimens were tested and the average value was reported for each sample. Deviations were less than ± 5%.

Thermal conductivity of the samples was measured using a TC3000E thermal conductivity tester (Xia xi electronic technology co. LTD, Xi’an, China) under 20 °C with a voltage of 0.80 V. The data of each sample was measured and collected every 3 min. The obtained thermal conductivity of each sample was the average of four values. Deviations were less than ± 3%.

The thermal degradation process of cotton and ramie fabric was probed by thermogravimetric analysis (TGA), which was performed using a TA Q5000IR thermo-analyzer (TA Instruments Inc., San Francisco, CA, USA) with a heating rate of 20 °C/min from room temperature to 800 °C in air atmosphere.

The combustion properties of the samples were evaluated using a cone calorimeter (Fire Testing Technology, Birmingham, UK) according to standard ISO 5660-1 [22]. The specimens were mounted on aluminium foil (with frame and no grid) and horizontally exposed to a cone heater with heat flux of 35 kW/m^2^. An ignitor was used before the samples were ignited. The sizes of cotton and ramie were 100 × 100 × 15 mm^3^ and 100 × 100 × 4 mm^3^.

## 3. Results

### 3.1. Optical Properties

**Figure 1:**
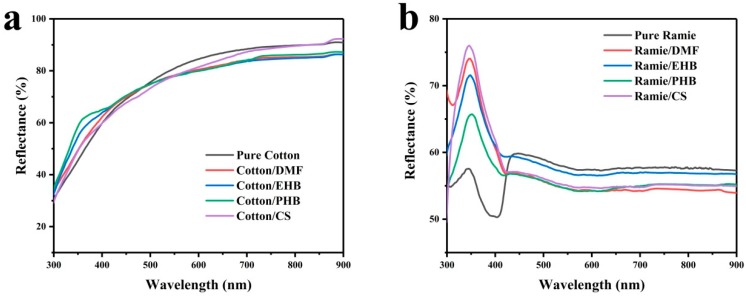
UV-Vis diffraction reflectance spectra of (**a**) cotton samples; (**b**) ramie samples.

UV-Vis diffuse reflectance spectroscopy was proved to be good at investigating the optical property of including cotton fabric [23]. In this work, the optical properties of treated cotton and ramie were studied through UV-Vis diffuse reflectance absorption spectra (Figure 1). All of the cotton samples exhibited similar increasing reflectance as the wavelength increased from 300 to 740 nm. The reflectance remained at around 90% for pure cotton and cotton/CS, 85% for cotton/EHB, cotton/PHB and cotton/DMF in the wavelength above 740 nm (Figure 1a). In the wavelength below 400 nm (ultraviolet light), the reflectance of the treated samples was higher than that of pure cotton. In the wavelength above 480 nm, all the treated samples showed lower reflectance than pure cotton. The addition of mold inhibitor increased the reflectance of cotton for ultraviolet light, the greatest increment was at wavelength of 361 nm where the reflectance of pure cotton and cotton/PHB was 48.8% and 61.6%, respectively. While the reflectance for visible light decreased by about 5% after the treatment of mold inhibitor.

The influence of mold inhibitor on the optical property of ramie showed similar tendency (Figure 1b). The reflectance of pure ramie had a peak (57.5%) at wavelength of 345 nm, then it dropped to 50.3% at wavelength of 404 nm followed by a sharp increase to the maximum of 59.8% at wavelength of 445 nm. As the wavelength increased, the reflectance tended to be steady at 57.5% from 540 to 900 nm. Each of the treated ramie curves had a peak at around 347 nm, the peak values were 65.7%, 71.5%, 74.0%, and 76.0%, much higher than that of pure ramie. The curves declined to 55%–60% at around 420 nm and then became stable at values of 54%–56% above the wavelength of 560 nm, lower than the pure ramie. The reflectance of ramie for ultraviolet light was greatly improved by adding the four kinds of mold inhibitors, which means that more UV light could be reflected rather than absorbed in treated samples. Meanwhile, reflectance of light in the wavelength above 440 nm was decreased, which means less visible light was reflected. The change of reflectance is attributed to the intrinsic optical properties of the four additives, all of which are crystal powder with UV-Vis DRS different from cotton and ramie fabrics. Therefore, one conclusion could be obtained that the introduction of mold inhibitors would slightly decrease the whiteness of cotton and ramie and limit the sterilizing effect of UV light.

### 3.2. Wettability

**Figure 2:**
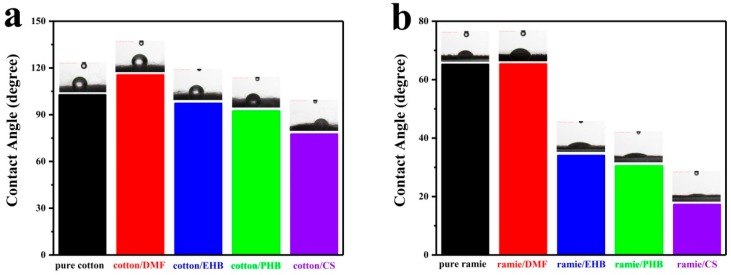
Contact angles of (**a**) cotton samples; (**b**) ramie samples.

Wettability is interrelated to hygroscopicity, which is one of the most valued properties in wearing. Humidity as an important factor in warehouse is also influenced by wettability. The wettability of cotton and ramie fabric was investigated by contact angle (CA) measurement. Deionized water was dropped on the surface of the sample. Testing results of cotton samples (Figure 2a) were taken at 20 s after the water was dropped. It is observed that the contact angle of cotton/DMF (115.51°) was higher than that of pure cotton (102.72°). Contact angles of cotton/EHB (97.24°), cotton/PHB (92.24°) and cotton/CS (77.49°) were lower than that of pure cotton. The wettability of material is basically decided by its surface energy and the roughness of its surface [24]. Modifying the surface roughness of textiles is normally achieved by loading inorganic particles through various methods [25]. Raw cotton has a relatively rough surface compared to its fabrics. Mold inhibitor additives in this work are organic small molecules which have little influence on changing the surface roughness of the cotton. [26] However, the additives were all fine crystal powders which had higher surface energy than that of raw cotton. The increased wettability of the treated cotton samples was mainly caused by the additives changing the surface energy. Additionally, the difference among all the treated samples can be attributed to the different surface energy of these additives.

Compared to raw cotton, ramie fabric is easier to be wetted as its surface is much smoother. One drop of deionized water permeated the pure ramie fabric in 10 s and ramie/DMF in 14 s. In the experiments of ramie/EHB, ramie/PHB, and ramie/CS, they were all permeated by a drop of water in only 2 s. Contact angles of all the ramie fabric samples were taken at 0.8 s after the water was dropped (Figure 2b). The wettability of ramie/EHB (CA = 34.02), ramie/PHB (CA = 30.43), and ramie/CS (CA = 17.24) was better than ramie/DMF (CA = 65.32) and pure ramie (CA = 65.20). The influences of the four additives on the wettability of raw cotton and ramie fabric were consistent, addition of EHB, PHB, and CS improved their wettability while DMF makes them harder to be wetted.

### 3.3. Thermal Conductivity

**Figure 3:**
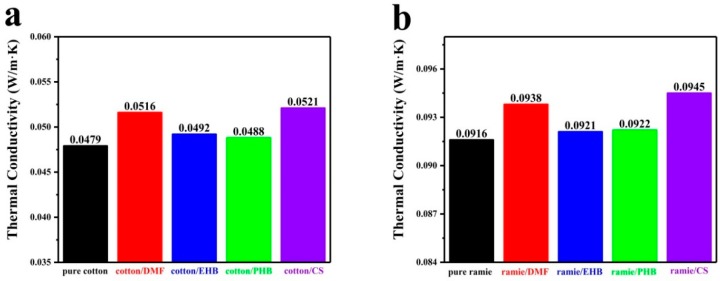
Thermal conductivity of **a**) cotton samples; **b**) ramie samples.

Thermal insulation and heat dissipation of clothes are directly related to the comfort for wearing. In addition, they are influenced by the thermal conductivity of the materials. Thermal conductivity of pure cotton and ramie and their mold inhibitor treated samples was measured by a TC3000E thermal conductivity tester (Figure 3). The thermal conductivity of pure cotton, cotton/DMF, cotton/EHB, cotton/PHB, and cotton/CS were 0.0479, 0.0516, 0.0492, 0.0488, and 0.0521 W/m·K (Figure 3a). As for the ramie fabric, the thermal conductivity of pure ramie, ramie/DMF, ramie/EHB, ramie/PHB, and ramie/CS were 0.0916, 0.0938, 0.0921, 0.0922, and 0.0945 W/m·K (Figure 3b). As observed in cotton and ramie fabric, the mold inhibitor treated samples exhibited an increment in thermal conductivity. This result can be attributed to the properties of the mold inhibitor. Both DMF and CS have a structure of vinyl group connected to the ester group forming conjugation effect which enhances heat transfer through electronic thermal conduction [27]. Additionally, the CS treated samples showed the highest thermal conductivity among all the treated samples, due to the 1, 4-butadienyl groups that also contribute to form a conjugation effect. The conjugation effect of paraben in EHB and PHB molecules was weaker than that of the two conjugation structures in DMF and CS, and the thermal conductivity increment of their treated samples was not so much as the other two samples. The increased thermal conductivity contributed to an improvement in heat dissipation and a decline in thermal insulation. A significant change of thermal conductivity for fabrics is usually achieved by large amount of loading thermally conductive additives with a good dispersion or even network structure [28,29]. However, none of the above conditions were met in this experiment, so it is plausible that the difference of thermal conductivities among all the samples was little. In general, the percentage of changed amount of thermal conductivity was less than 9%.

### 3.4. Thermal Stability and Combustion Properties

**Figure 4:**
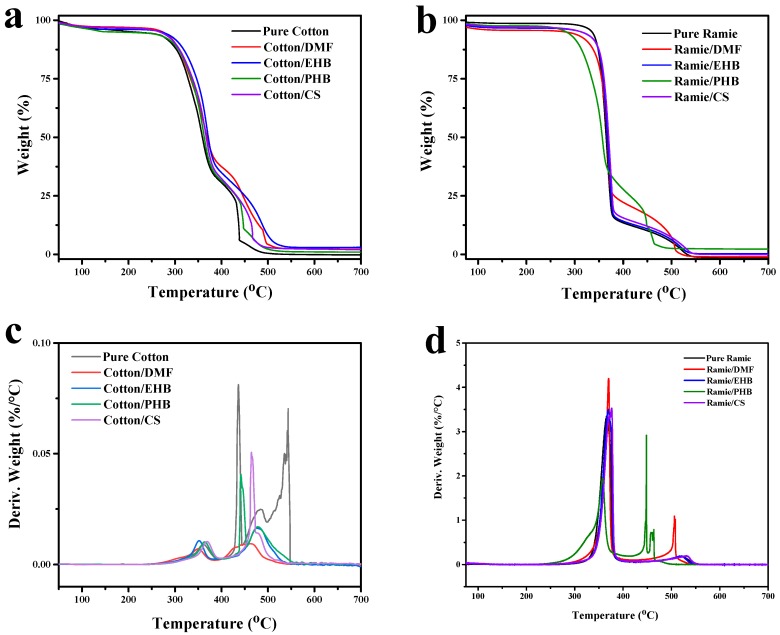
Thermal degradation curves of (**a**) cotton samples; (**b**) ramie samples. In addition, derivative weight of (**c**) cotton samples; (**d**) ramie samples.

Thermogravimetric analysis (TGA) was used to study the thermal stability of cotton and ramie fabric (Figure 4). The pure cotton sample had lost 5% of its weight at the temperature of 266 °C, mainly caused by loss of absorbed moisture and low molecular weight compounds (Figure 4a,c). A 55% weight loss that happened from 298 to 385 °C can be attributed to the dehydroxylation, carbonization, and decomposition of the cellulose chains. Another sudden drop of weight from 25% to 6% in the temperature range from 430 to 440 °C was found due to the destruction and pyrolysis of carbonized char residues. The treated cotton samples showed similar degradation curves from 50 to 380 °C, while they remained a bit more weight percentage than pure cotton from 380 to 500 °C, indicating higher density of carbonized char was formed in these treated samples.

The initial degradation temperature of pure ramie fabric was higher than that of cotton, because the main composition of ramie fabric is bast fiber which is intrinsically stronger with higher crystallinity and orientation. [30] Degradation of ramie fabric also had a sharp decline in the temperature range from 330 to 380 °C (Figure 4b,d) as the majority of its chemical composition is cellulose, the same as cotton. Decomposition of hemicellulose and pectin in ramie fabric also happened in this stage. Mass loss at a temperature higher than 380 °C is mainly caused by the oxidative degradation of carbonized chars. [31] Ramie/EHB and ramie/CS had TGA curves similar to that of pure ramie. The initial degradation temperatures of ramie/DMF (T_−5%_ = 280 °C) and ramie/PHB (T_−5%_ = 282 °C) were lower than the other samples. This is because the thermal stability of DMF and PHB were not as good as the others, the vaporization and decomposition of them happened at lower temperature and flammable gas produced in this stage facilitated the dehydroxylation, carbonization, and decomposition of cellulose. However, the remained weight percentage of ramie/PHB and ramie/DMF from 380 to 450 °C were higher than the other samples, indicating the form of more char residues in these two samples. This can be explained by a proposed pyrolysis process that the weight loss at lower temperature was mostly attributed to carbonization of polymer chains which forms char residues.

**Figure 5:**
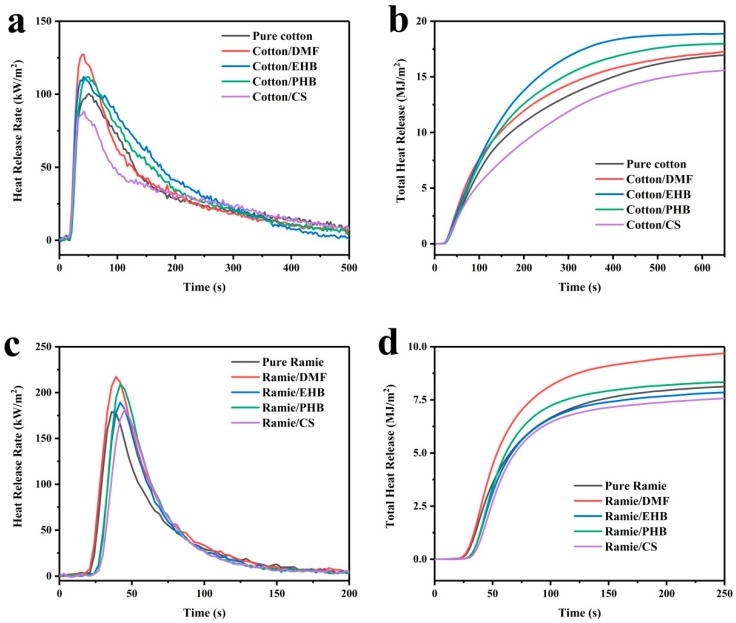
Heat release rate of (**a**) cotton samples; (**c**) ramie samples; total heat release of (**b**) cotton samples; (**d**) ramie samples.

Cone calorimeter is an effective measurement of combustion property for most materials [32,33], including textiles [34]. Combustion property of cotton and ramie fabrics was measured by a cone calorimeter (Figure 5). After ignition by 33 s, the heat release rate of the cotton sample reached a peak of 100.4 kW/m^2^ (Figure 5a), then the HRR dropped to 50 kW/m^2^ 85 s later. The fire lasted for 385 s and its total heat release (THR) was 16.9 MJ/m^2^ (Figure 5b). The cotton/DMF, cotton/EHB, and cotton/PHB samples showed higher pHRR (127.3, 111.3, and 111.8 kW/m^2^) and THR (18.8, 18.0, and 17.3 MJ/m^2^) than pure cotton. The boiling point of the three additives (DMF at 193 °C, EHB at 297 °C, PHB at 133 °C) were lower than the 300 °C. As investigated before by TGA, the decomposition of the cellulose chains in the cotton samples mainly happened at temperature above 300 °C. It is deduced that the additives have already evaporated to flammable gas when the cotton is about to burn, making it burn more quickly and thoroughly thus reaching a higher pHRR. Different from the other three additives in this work, calcium sorbate is stable under temperature below 400 °C and one of its pyrolysis product-calcium carbonate can be used as a fire retardant filler [35,36]. The pHRR (88.5 kW/m^2^) and THR (15.6 MJ/m^2^) of cotton/CS were lower than that of pure cotton samples.

**Figure 6:**
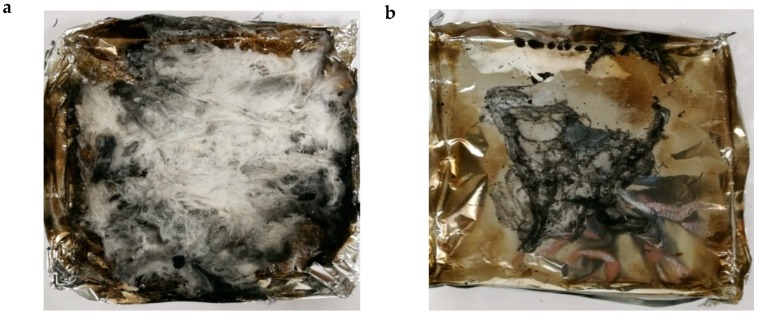
Char residues of (**a**) cotton/calcium sorbate (CS); (**b**) ramie/CS after cone calorimeter test.

All the pHRR of treated ramie fabric were higher than that of pure ramie (Figure 5c). The highest pHRR was 217.2 kW/m^2^ of ramie/DMF, 21.4% higher than that of pure ramie. Unlike the result obtained from raw cotton test, the addition of calcium sorbate did not show any fire retardant effect in the ramie fabrics. This is because macroscopic forms of raw cotton and ramie fabrics differ a lot. The squeezed raw cotton has lots of inner parts isolated from the air which did not burn out (Figure 6a) and it is easier to form carbonized chars with the addition of fire retardant fillers. In contrast, most parts of woven fabrics are exposed to air, thus it easily burnt out (Figure 6b) and only flame retardant coatings with enough amounts or multicomponent can effectively inhibit its burning [34,37]. The amount of added calcium sorbate in the ramie fabric for mildew proof was not enough for flame retardancy. The HRR and THR curves of ramie/DMF and ramie/PHB did not accord with their TGA curves. Since the experimental conditions of the two tests are different. Specimens were gradually heated from room temperature to high temperature, went through all temperature stages in TGA test, while in cone calorimeter tests, they were exposed to a cone radiative heater with the power of 35 kW/m^2^ during the entire process.

## 4. Conclusions

In this work, the optical property, wettability, thermal conductivity, thermal, and combustion properties of raw cotton and ramie fabric treated by four kinds of mold inhibitors (dimethyl fumarate, ethyl p-hydroxybenzoate, propyl p-hydroxybenzoate, and calcium sorbate) were investigated. Results show that the reflectance of UV light has been increased due to the intrinsic optical properties of the additives. The addition of EHB, PHB, and CS improved the wettability of raw cotton and ramie fabric while DMF makes them harder to be wetted, which is attributed to the different surface energy of these additives. Thermal conductivity was slightly increased in an extent of changing less than 9%. These additives did not make a big difference in the pyrolysis process of cotton and ramie fabric. The addition of DMF, EHB, and PHB caused 26.8%, 11.0%, and 11.3% increase in pHRR, and 11.2%, 6.5%, and 2.4% increase in THR for cotton samples owing to the flammability of the small molecular additives. While the addition of CS caused 11.9% decrease in pHRR and 7.7% decrease in THR for cotton thanks to the flame retardancy of CS. PHRRs of all treated ramie fabric and THRs of ramie/DMF and ramie/PHB increased. This work studied the influence of four commonly used mold inhibitors on optical property, wettability, thermal conductivity, thermal stability, and combustion properties of raw cotton and ramie fabric, which are main influencing factors for their warehousing and further application. The results may be of interest to those who transport, store, or process textiles.
